# Mistaken Identity: Clarification of *Rubus coreanus* Miquel (Bokbunja)

**DOI:** 10.3390/molecules190710524

**Published:** 2014-07-18

**Authors:** Jungmin Lee, Michael Dossett, Chad E. Finn

**Affiliations:** 1 United States Department of Agriculture (USDA), Agricultural Research Service (ARS), Horticultural Crops Research Unit (HCRU) Worksite, Parma, ID 83660, USA; 2 BC Blueberry Council (in partnership with Agriculture and Agri-Food Canada-Pacific Agri-Food Research Centre), 6947 Hwy #7, P.O. Box 1000, Agassiz, BC V0M 1A0, Canada; E-Mail: michael.dossett@agr.gc.ca; 3 United States Department of Agriculture, Agricultural Research Service, Horticultural Crops Research Unit (HCRU), Corvallis, OR 97330, USA; E-Mail: chad.finn@ars.usda.gov

**Keywords:** species adulteration, cha tian pao, bramble, caneberry, blackcap

## Abstract

In the U.S., there has been a recent surge in Korean black raspberry products available and in the number of reports about this species appearing in the scientific literature. Despite this, the majority of products sold and the work carried out has been on *Rubus occidentalis* L., not *R. coreanus* Miquel. The importance of accurate recognition of all starting material is multiplied for research downstream, including genetics/genomics, plant breeding, phenolic identification, food processing improvements and pharmacokinetic investigations. An overview of distinguishing characteristics separating *R. coreanus* from *R. occidentalis* will be presented. Research conducted on correctly identified fruit will also be summarized to aid future studies that might showcase the unique qualities that bokbunja can offer.

## 1. Introduction

According to the 1867 records of Friedrich Miquel [1], wild *Rubus coreanus* Miq. (bokbunja native to eastern Asia) Chinese, Japanese and Korean [2] plants and fruit were collected in Korea by Richard Oldham and verified by Naohiro Naruhashi, as early as 1863. Within the *Rubus* genus, *R. coreanus* is in the subgenus, *Idaeobatus*, along with at least 99 other *Rubus* species, including other commercially harvested species, such as red raspberry (*R. idaeus* L.), the Japanese wineberry (*R. phoenicolasius* L.), the Andean blackberry (*R. glaucus* Benth.), Mysore raspberry (*R. niveus* Thunb.) and the black raspberry (*R. occidentalis* L.) [2]. In the late 1960s, commercial cultivation of what was thought to be *R. coreanus* (anonymous Korean commercial grower) started in South Korea. While *R. coreanus* (bokbunja) beverage products were marketed as traditional foods, they were unlike a true Korean traditional food (e.g., kimchi) in that they were not readily available in the marketplace until around the year 2004 (personal observation; [3]). A recent literature search showed an increase in *R. coreanus* research articles being published around the year 2007.

Identity concerns over *R. coreanus* plants [3,4,5,6] were initially brought to our attention from the fruit images utilized on bokbunja commercial products in the U.S. marketplace; *R. coreanus* (Korean black raspberry) fruit was misrepresented by images of *R. occidentalis* (native to eastern North America, [2]) fruit. Only a small fraction of commercially cultivated black raspberries in Korea are *R. coreanus*, while the majority (reported at >2,800 hectares in 2013 cultivated by >10,000 farmers; [3,7,8]) are actually *R. occidentalis* (personal observation; anonymous Korean commercial grower; [3,4,5,8]). Based on randomly amplified polymorphic DNA fragments and chloroplast markers, Eu *et al.* [3,4,5] demonstrated that commercially grown black raspberry plants in Korea are more closely related to North American *R. occidentalis* cultivars than to native *R. coreanus* and, in fact, are *R. occidentalis* not *R. coreanus*. Currently, production of *R. coreanus* in Korea is unable to meet the demand for bokbunja products. Identifying the best *R. coreanus* selections or breeding cultivars for commercial plantings is underway by Kim *et al.* [8,9], where Kim *et al.* [9] already has identified promising cultivars (Jungkeum 1, Jungkeum 2, Jungkeum 3, Jungkeum 4 and Jungkeum 5).

Phenolic profiles have become a valuable laboratory tool in small fruit research: our own studies of species, cultivar and genotype in blueberries (*Vaccinium corymbosum* L., *V. deliciosum* Piper, *V. membranaceum* Douglas ex Torr., *V. ovalifolium* Sm. and *V. ovatum* Pursh.), strawberries (*Fragaria* spp. L.), elderberries (*Sambucus canadensis* L. and *S. nigra* L.), black raspberries (*R. occidentalis* and *R. coreanus*) and lingonberries (*V. vitis-idaea* L.) were greatly aided by the ability to contrast phenolic profiles [10,11,12,13,14,15,16,17,18,19,20,21]. This collective phenolic literature directly assists ingredient assurance and product quality control and can be used in authenticity and adulteration monitoring, phenolic degradation, pharmacokinetics, *etc.,* but when misidentified fruit (thought to be that of *R. coreanus*) is harvested, all work downstream becomes misinformation that only causes further disorder. For example, our *Rubus* phenolic review article [22] was written before access to authenticated *R. coreanus* fruit samples existed [6], and it summarized some scientific papers that were conducted on incorrectly identified *R. coreanus* fruit. The health benefits of *R. coreanus* fruit might be uniquely different from *R. occidentalis*, but this is difficult to gauge based on the current confusion among growers, producers and scientific communities.

A one-page fact-sheet with photos depicting leaves, flowers, fruit and anthocyanin profiles is available for download to help growers, ingredient suppliers, food processors, and researchers distinguish between these two black raspberries [23]. The objective of this review is to reduce future mistakes by highlighting this issue, to provide a guide to clearly differentiate these species and to provide a summary of phenolic research conducted on the actual *R. coreanus* fruit.

## 2. History of Commercialization of *Rubus coreanus* and *R. occidentalis* Plants

*Rubus occidentalis* has been widely grown commercially in eastern North America, where it is native, since the mid-late 1800s [24] and has been used in a variety of food products because of its dark color and unique flavor [22]. While *R. coreanus* is not cultivated commercially in North America, as early as 1937, Darrow [25] recognized its value as a source of resistance to a variety of disease pathogens for breeding. Unfortunately, this potential has not been fully realize; while *R. coreanus* has been valuable in breeding red raspberry [26], hybrids with *R. occidentalis* are highly sterile [27]. It is not clear when *R. occidentalis* was first introduced to Korea. We are unaware of any work comparing the agronomic qualities of these two species as grown in Korea; however, in North America, *R. coreanus* is vigorous and resistant to many of the diseases that cause problems for black and red raspberry growers. Despite this, its fruit tends to be smaller and softer and lack the distinctive flavor of *R. occidentalis*. These reasons, combined with its vigor and thornier canes that may make *R. coreanus* more difficult to manage, could be part of the reason why it is not as commonly grown on a commercial scale.

## 3. Morphological and Phenological Differences

**Figure 1:**
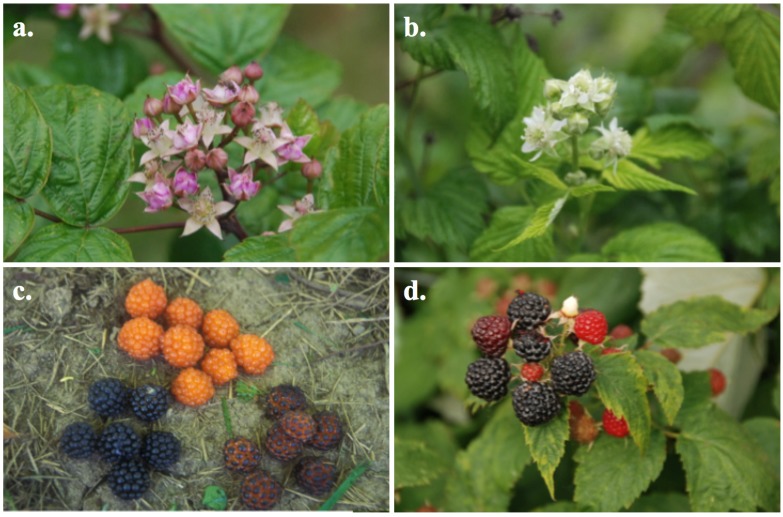
There are clear distinguishing morphological differences between *Rubus coreanus* and *R. occidentalis*. A photo of leaves can be found in Lee *et al.* [6]. Again, *R. coreanus* has pink flowers (**a**) and appears glossy, as there is less white hair (pubescence) on the fruit (**c**). *Rubus occidentalis* has white flowers (**b**) and white hair on the fruit (**d**), which make the fruit appear dull.

*Rubus coreanus* flowers are a light to dark purple-pink color [3,6,8,28] compared to the white colored flowers of *R. occidentalis*. *Rubus coreanus* plants typically have two or more additional leaflets compared to *R. occidentalis*; *R. coreanus* typically has five to nine leaflets that are always pinnately-arranged, while *R. occidentalis* usually has three (ternate) or occasionally five palmately-arranged leaflets (Lee *et al.* [6]). *Rubus coreanus* fruit are superficially similar to those of *R. occidentalis*; genotypes of both species produce fruit that ranges from albino (orange), purple to black in color, and the fruit is hollow, as the torus remains on the plant when the fruit is picked [6]. However, well-formed fruit of *R. occidentalis* have smaller drupelets, leading to a smoother surface contour, and usually have some degree of fine white pubescence. This pubescence may occur across the epidermis of the *R. occidentalis* fruit, but is usually concentrated around the edges of the drupelets and is less evident in *R. coreanus*, leading to a somewhat glossier appearance. Fruit of *R. coreanus* can have an unusual bicolored appearance, where anthocyanins concentrate into dark spots on the tip of each drupelet, at the base of the style, against an orange background (see Figure 1c; orange with dark spots on the top of each drupelet of aggregate fruit). Clear images of the leaves, flowers and fruit can be found in Lee *et al.* [6], Eu *et al.* [3,4], Kim *et al.* [9] and in Figure 1. Plant size, vigor, leaf morphology, cane morphology and fruit ripening dates can be found in Lee *et al.* [6], Keep *et al.* [28] and Miquel [1]. *Rubus coreanus* fruit ripen in late July and early August, whereas *R. occidentalis* fruit ripen a few weeks earlier (in June/July) [6,8].

## 4. Anthocyanin Profiles

**Figure 2:**
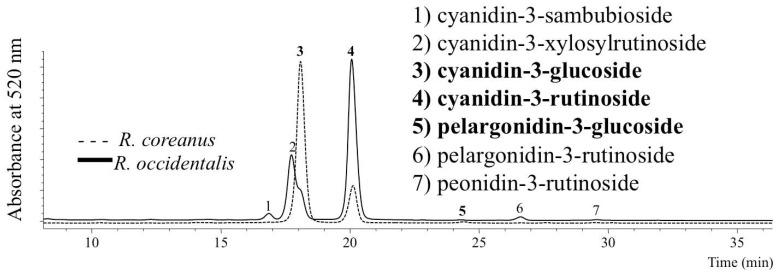
Anthocyanin profile of *Rubus occidentalis* cv. Munger (solid line) and *R. coreanus* (dotted line) fruits. Anthocyanin peak identifications in bold are the ones found in *R. coreanus* fruit [6,10].

Besides their unique vegetative traits, the two species have distinctive anthocyanin profiles (Figure 2 and Table 1). *Rubus coreanus* fruit contains fewer anthocyanins (up to three) compared to *R. occidentalis* (up to seven) [6,10,11,12,18,19,20,21]. A list of the individual anthocyanins can be found in Table 1. A clear anthocyanin profile of ‘Munger’ fruit overlaid with *R. coreanus* fruit is shown in Figure 2. In the U.S., the cultivar, Munger (*R. occidentalis*), is the most widely grown, and ‘Munger’ fruit has a reliable anthocyanin profile over varying growing seasons (comparing Figure 2 to Dossett *et al.* [18,19]). While both species contain glycosides of cyanidin and pelargonidin [6,10], trace levels of peonidin-3-rutinoside are only reported in some *R. occidentalis* fruit [18,19,20,21].

Our findings [6,10] confirm the identification correctly reported by Kim *et al.* [29], Heo *et al.* [30] and Lee *et al.* [31]. The two anthocyanins Kim *et al.* [29] found in *R. coreanus* fruit were glucoside and rutinoside of cyanidin, and cyanidin-3-rutinoside was the main pigment, followed by cyanidin-3-glucoside. In samples from CRUB 1634 16-1 fruit (*R. coreanus* genotype at USDA-ARS), cyanidin-3-rutinoside (lightest colored fruit) was also the chief anthocyanin, though fruit from two other *R. coreanus* genotypes (CRUB 1634 19-28 and CRUB 1634 19-23) from the same population had more cyanidin-3-glucoside and less cyanidin-3-rutinoside [6]. Heo *et al.* [30] and Lee *et al.* [31] also reported only two measurable anthocyanins in *R. coreanus*. Heo *et al.* [30] described cyanidin-3-rutinoside content being greater than cyanidin-3-glucoside in mature fruit, but found the order reversed in immature fruit. Since *R. coreanus* does not contain xylose-containing pigments (*i.e.*, cyanidin-3-xylosylrutinoside and/or cyanidin-3-sambubioside; see Figure 2. and Table 1), their detection indicates the presence of *R. occidentalis* fruit or another unknown contaminant and that the sample is not pure *R. coreanus*.

**Table 1:** Anthocyanins found in *Rubus coreanus versus R. occidentalis* fruit. Anthocyanins listed in the order of HPLC elution. ‘+’ indicates present. ‘−’ indicates not present. ‘+/−’ indicates both cases have occurred. A clear recent example of additional anthocyanin profiles of the two species can be found Lee *et al.* [6], Dossett *et al.* [18] and Lee [10,11,12].

Peak Numbering in Figure 2.	Anthocyanin	*R. coreanus*	*R. occidentalis*
1	cyanidin-3-sambubioside	−	+
2	cyanidin-3-xylosylrutinoside	−	+ *
3	cyanidin-3-glucoside	+	+
4	cyanidin-3-rutinoside	+	+/−
5	pelargonidin-3-glucoside	+/−	+/−
6	pelargonidin-3-rutinoside	−	+/−
7	peonidin-3-rutinoside	−	+/−

* Cyanidin-3-xylosylrutinoside was found lacking in the fruit of one wild collected *R. occidentalis* plant out of >1,000 genotypes analyzed in our laboratory [6,10,11,12,18,19,20,21]. Lacking cyanidin-3-xylosylrutinoside in *R. occidentalis* fruit occurs rarely [20].

Due to this difference in the anthocyanin profile (chemotaxonomical distinction), products from these species can be identified in the absence of the vegetative attributes described above. For example, a Korean commercial bokbunja juice sample was obtained, and analysis showed that it had the anthocyanin profile of *R. occidentalis* fruit, not *R. coreanus* fruit [10]. This commercial juice contained cyanidin-3-sambubioside and cyanidin-3-xylosylrutinoside, not found in *R. coreanus*. Researchers should be aware that after processing (*i.e.*, freeze drying, juicing, concentrating, heating), the proportion of the individual anthocyanin peaks might be altered, and unknown polymeric anthocyanins may be formed and appear in the chromatograms, as pointed out by Lee *et al.* [32], Lee and Wrolstad [33], Lee [11], Sadilova *et al.* [34] and Novotny *et al.* [35]. Techniques for improved retention of color using food processing methods, ideal storage condition, *etc*., will result in differing response between *R. coreanus* and *R. occidentalis*, since the predominant cyanidin-based anthocyanins in their fruits have different colors, tinctorial strengths (visual detection threshold), spectral characteristics, thermal degradation kinetics, *etc.*, due to independent structures [34,35,36]. Different cyanidin-based anthocyanins exhibit altered bioavailability in human subjects [37,38,39], so the potential health benefits of *R. coreanus* fruit might be unique and different from *R. occidentalis*, as the dominant anthocyanin and the ratio of the individual anthocyanins are characteristic for each species.

## 5. Phenolics Other Than Anthocyanins

From published bokbunja data, two studies that worked with correctly identified *R. coreanus* fruit [30,31] have reported the non-anthocyanin phenolic profile in *R. coreanus* fruit. Phenolic acids (ellagic acid and coumaric acid hexose), flavonol-glycosides (quercetin-glucoside, quercetin-rutinoside, quercetin-glucuronide and kaempferol-glucoside), flavanol polymers (numerous procyanidins; tentatively identified) and hydrolyzable tannins (numerous ellagic acid derivatives; tentatively identified) are in *R. coreanus* fruit [30,31]. It is certain that *R. coreanus* fruit contains ellagic acid derivatives [30,31], since they are widely distributed in *Rubus* fruit [22], but that group of phenolics remains challenging to identify and quantify [22].

*Rubus occidentalis* fruit has been reported to contain the same non-anthocyanin phenolic classes as *R. coreanus*, but with some differences in the individual phenolics within: phenolic acids (ellagic acid, ferulic acid, caffeic acid, *p-*coumaric acid, dihydroxybenzoic acid, *etc.*), flavonol-glycosides (quercetin-glucoside, quercetin-rutinoside, myricetin-glucoside, dihydrokaempferol-glucoside), flavanol monomers (epicatechin) and hydrolyzable tannins (numerous ellagic acid derivatives) [22,39,40].

Phenolics other than anthocyanins in *R. coreanus* and *R. occidentalis* fruits remain a much-needed area of research [22]. Due to the lack of available non-anthocyanin phenolic standards (especially for the larger compounds, like ellagitannins), and the challenges to extract, isolate and analyze these compounds [22], utilizing anthocyanin profiles for authenticity and adulteration is easier and clearer [11,16,41,42]. Examples of using anthocyanin for the authenticity of fruit products, cranberry (*V. macrocarpon* Ait.) juice and *R. occidentalis* fruit sold as dietary supplements are provided in Lee [11,16]. Again, randomly amplified polymorphic DNA fragments and other genetic markers can also be used to distinguish these two species, as illustrated by Eu *et al.* [3,4].

## 6. Studies Reporting on the Incorrect Species

The unique phytochemical composition (specifically anthocyanin), as explained above, is the principal reason why it is crucial that bokbunja processing, storage and pharmacokinetic work be done on the correct species, especially if companies or researchers hope to find that *R. coreanus* fruit and products offer exclusive benefits compared to the more widely available *R. occidentalis*; otherwise, our knowledge of *R. coreanus* fruit benefits will only add to the findings of consuming *R. occidentalis* fruit or potentially create confusing and/or conflicting results. A list of incorrectly identified *R. coreanus* fruit used in further research was summarized before [10], though the three examples below emphasize the misunderstandings created from incorrectly identifying the subject species. An interesting note is that Examples 2 and 3 obtained samples from commercial fields and food processors.

(1) Hyun *et al.* [7] actually reports on the anthocyanin biosynthetic genes involved in *R. occidentalis*, not *R. coreanus* fruit, despite what is reported in the paper. In the fruit image provided by Hyun *et al.* [7], the pubescence on the aggregate fruit is clearly present, and they report the presence of cyanidin-3-xylosylrutinoside, which is an indicator that these fruits are that of *R. occidentalis*, not *R. coreanus*. This study [7] examined cultivated black raspberry from Gochang, Korea.

(2) Ku and Mun [43] used black raspberry liquor (cordial) press cake (from commercial liquor processor, Gochang, Korea) for additional phenolic extractions in value-added product development, but the extraction optimizations were conducted on *R. occidentalis* press cakes, not *R. coreanus*, as indicated by the presence of cyanidin-3-sambubioside and cyanidin-3-xylosylrutinoside, which are not found in *R. coreanus* fruit.

(3) Kim *et al.* [44] used misidentified *R. coreanus* fruit to conduct a phytochemical identification study (reported cyanidin-3-sambubioside presence, which is not found in *R. coreanus* fruit), then used those fruit to conduct a study on whether these (misidentified *R. coreanus*) fruit could aid in reducing DNA damage to cigarette smokers [45]. This study [44] obtained samples from a commercial field from Gokseong, Korea.

## 7. Conclusions

Most cultivated *R. coreanus* fruit in Korea are that of *R. occidentalis* based on vegetative traits, fruit anthocyanin profiles and DNA profiling, as discussed above. Commercial bokbunja product ingredient listings need to be corrected to *R. occidentalis* to prevent further confusion. Since there is nothing wrong with growing *R. occidentalis* in Korea for the functional food market, we only propose that the correct species name is utilized on labeling and documentation to prevent confusion in the marketplace and research community. We are hopeful that future work on *Rubus* fruit will be clear, whether *R. coreanus*, *R. occidentalis* or a mix of the two is used. It is helpful to have the fruit authenticated by a well-trained plant taxonomist prior to further examining its processing stability, health benefits, *etc.* Genetic fingerprinting has become a relatively inexpensive service provided by commercial laboratories, and the information produced by Eu *et al.* [3,4] would allow any of these laboratories to confirm which species they are using in their study. If a well-trained taxonomist is not available, then this review article and several papers referenced in this work will provide guidance for clear identification.
